# The Role of Non-Coding RNA in Congenital Heart Diseases

**DOI:** 10.3390/jcdd6020015

**Published:** 2019-04-01

**Authors:** Angel Dueñas, Almudena Expósito, Amelia Aranega, Diego Franco

**Affiliations:** Cardiovascular Development Group, Department of Experimental Biology, University of Jaen, 23071 Jaen, Spain; aduenas@ujaen.es (A.D.); aev00006@red.ujaen.es (A.E.); aaranega@ujaen.es (A.A.)

**Keywords:** non-coding RNA, microRNAs, lncRNAs, congenital heart disease

## Abstract

Cardiovascular development is a complex developmental process starting with the formation of an early straight heart tube, followed by a rightward looping and the configuration of atrial and ventricular chambers. The subsequent step allows the separation of these cardiac chambers leading to the formation of a four-chambered organ. Impairment in any of these developmental processes invariably leads to cardiac defects. Importantly, our understanding of the developmental defects causing cardiac congenital heart diseases has largely increased over the last decades. The advent of the molecular era allowed to bridge morphogenetic with genetic defects and therefore our current understanding of the transcriptional regulation of cardiac morphogenesis has enormously increased. Moreover, the impact of environmental agents to genetic cascades has been demonstrated as well as of novel genomic mechanisms modulating gene regulation such as post-transcriptional regulatory mechanisms. Among post-transcriptional regulatory mechanisms, non-coding RNAs, including therein microRNAs and lncRNAs, are emerging to play pivotal roles. In this review, we summarize current knowledge on the functional role of non-coding RNAs in distinct congenital heart diseases, with particular emphasis on microRNAs and long non-coding RNAs.

## 1. Introduction

Cardiovascular development is a complex developmental process involving the formation and alignment of distinct cell sources. Early cardiogenesis starts soon after gastrulation as symmetrical precardiogenic crescents are formed at both sides of the developing embryo [1]. Left and right crescents converge into the embryonic midline forming the early cardiac straight tube. At this stage, the entire embryonic heart is composed of two distinct layers, an external myocardium and internal endocardium. Soon thereafter, the developing heart initiates a rightward looping, configuring prospective atrial and ventricular chambers, although it remains to be a single tube [2,3]. Subsequently, mesenchymal components are developed within specific regions of the embryonic heart forming the anlage of the future cardiac valves. Left and right components are delineated on each cardiac chamber by a complex process of septation, generating thereafter a four-chambered heart with distinct inlet and outlet connections. In addition to these large macroscopic changes, subtler but similarly important changes occur in the embryonic and fetal heart. An external epicardial layer is formed, which is derived from the proepicardium [4] and cardiac neural crest migrate into the anterior pole of the heart providing cellular cues to govern aortico-pulmonary septation [5]. In addition, embryonic ventricular trabeculations are progressively remodeled [6,7,8], configuring internally a thinner trabecular network and externally a thicker compact layer.

Given the complexity of morphogenetic processes orchestrating cardiac development, impairment in any of these developmental events invariably leads to cardiac defects. Over the last decades, our understanding of the developmental defects causing cardiac congenital heart diseases (CHD) has exponentially increased and culprit developmental tissues have been identified (see for recent reviews [9,10]). For example, impaired cardiac neural crest migration leads to abnormal arterial pole development, ranging from absence, i.e., permanent truncus arteriosus, to defects on aortic-pulmonary septation such as double outlet right ventricle (DORV) [5]. Impaired valve development is also frequently associated with VSDs, as the membranous part of the septum fails to converge properly [11,12,13]. Abnormal left/right symmetry is at the bases of heterotaxia, atrial isomerism and also double inlet left ventricle [3]. In addition, careful examination of complex CHDs such as Tetralogy of Fallot (TOF) has also provided evidence of simultaneous impairment of multiple cardiac embryonic structures.

CHD is defined as clinically structural heart defect present before and/or at birth and is the leading cause of infant morbidity and mortality worldwide, with an incidence of 1–2% in newborns [14]. CHD can be classified into three broad categories according to their clinical manifestations: cyanotic heart disease, left-sided obstruction defects, and septation defects [15]. Cyanotic heart diseases include TOF, transposition of the great arteries (TGA), tricuspid atresia, pulmonary atresia, Ebstein’s anomaly of the tricuspid valve, DORV, persistent truncus arteriosus (PTA) and total anomalous pulmonary venous connection. Left-sided obstructive lesions, the second main type of CHDs, include hypoplastic left heart syndrome (HLHS), mitral stenosis, aortic stenosis, aortic coarctation and IAA. The third main type of CHDs are septation defects, affecting separation of the atria (atrial septal defects, ASDs), the ventricles, (ventricular septal defects, VSDs) or both (atrioventricular septal defects, AVSDs) [16].

The molecular culprits of CHD pathogenesis remain rather unclear. The advent of the molecular era started to bridge morphogenetic with genetic defects. Our current understanding of the transcriptional regulation of cardiac morphogenesis has enormously increased. Key transcription factors such as *NKX2.5*, *GATA4* and *MEF2C* are essential for early stages of cardiac morphogenesis [1]. *PITX2* is essential for left/right patterning [3,17] and multiple members of the T-box family play pivotal developmental roles in distinct compartments of the developing heart [18]. Importantly, the impact of these transcription factors in cardiac CHD has been obtained by two complementary approaches. Genetic manipulation of experimental animals provided invaluable tools to dissect the functional roles of a large set of transcription factors in cardiogenesis. For example, genetic deletion of *PITX2* have unraveled a key role for this transcription factor in atrial isomerism [19]. In addition, the rapid advance of genetic screening of CHDs familiar cases and more recently large cohorts of similarly affected CHD patients has greatly increased our understanding of the genetic and molecular bases of CHD [16]. These efforts have identified, for example, *TBX1* as culprit gene underlying DiGeorge syndrome [20,21]. However, despite major efforts dissecting the genetics of CHDs, they only account for only <20% of all diagnosed cases [9,10]. Thus, novel agents remain to be discovered and currently two sets of evidence are put forward: discovering the impact of environmental agents to genetic cascades [22,23,24,25] and novel genomic mechanisms regulating gene expression such as post-transcriptional regulatory mechanisms [26,27,28,29].

Over the last decade, we have witnessed a new revolution on the understanding of genome biology. Protein coding genes only account for approximately 2–3% of the genome, whereas the rest of the genome was considered “dark matter” or even “trash DNA”. The discovery that 60–70% of the “dark matter” genome is indeed transcribed but not translated has completely modified our vision of genome biology and thus of gene regulatory mechanisms (see for a recent review [30]). Non-coding RNAs can be grossly subclassified into two major groups: small non-coding RNAs (shorter that 200 nucleotides in length) and long non-coding RNAs (longer that 200 nucleotides in length). Small non-coding RNAs contain distinct subclasses such as piwi-RNA, snoRNAs, siRNA and the most numerous and well-studied group of microRNAs [31,32]. microRNAs are small non-coding RNAs of 22–24 nucleotide that are capable of regulating gene expression by interacting with mRNA transcript 3´UTRs, promoting thereafter mRNA degradation and/or protein translation blockage [33]. Long non-coding RNAs are an equally heterogenous group of non-coding RNAs that are basically subclassified according to their functional properties yet such a classification is complex and imprecise given our limited understanding of their function [34,35]. Furthermore, complex interplays between long non-coding RNAs and microRNAs are also reported [36,37], opening a completely novel landscape to transcriptional and post-transcriptional regulation of gene expression.

In this review, we summarize current knowledge of the impact of non-coding RNAs in the development of distinct CHDs, with particular emphasis on the role of microRNAs and long-non-coding RNAs when available.

## 2. microRNAs and Cardiac Congenital Heart Diseases

microRNAs are small (22–24 nucleotides in length) non-coding RNAs that modulate protein function by binding to target messenger RNA, resulting in repression of protein translation and/or mRNA degradation. Experimental cardiac-specific disruption of *DICER*, an RNase that is essential for microRNA processing and biosynthesis [38,39], leads to embryonic lethality within first four days after birth due to dilated cardiomyopathy in mice [40]. This study therefore highlights the importance of microRNAs during embryogenesis and cardiac development. Although *DICER* deletion revealed a crucial role for those small RNAs in cardiac development in vivo, no specific microRNA deletion has yet led to a fully penetrant lethal phenotype in mice, thus demonstrating complex microRNA actions of and plausible compensatory mechanisms. Nonetheless, an important number of microRNAs have been shown to play key roles in heart function [41,42,43,44,45,46,47,48] and the most relevant microRNA leading to CHDs is miR-1 [41,47]. miR-1 targets the cardiac transcription factor *HAND2*, which is involved in embryonic heart growth, as well as several other regulators of cardiac growth during development. Deletion of miR-1 in mice results in complex heart defects including VSDs. Surviving miR-1 deficient mice have conduction system defects and increased cardiomyocyte proliferation. Hence, miR-1 dysregulation might result in CHD in humans [16]. In addition to experimental evidence, microRNAs are attractive clinical biomarkers as they remain stable in blood, urine and other biological fluids and evade RNA degrading enzymes. In this context, Yu et al. [49] hypothesized, and Zhu et al. [50] demonstrated, that microRNAs in maternal serum are candidate biomarkers for prenatal detection of fetal CHD in early pregnancy. These authors identified four significantly up-regulated microRNAs (miR-19, miR-22, miR-29c and miR-375) in mothers carrying fetuses with CHD, being miR-19b and miR-29c significantly up-regulated in VSDs and all four microRNAs up-regulated in TOF. These results are very important because they suggest that specific microRNA might be associated to distinct types of CHD. In the following sections, we provide a systematic review of the current understanding of the functional role of microRNAs on the pathogenesis of cardiac CHD within the three major CHD subtypes, cyanotic heart disease, left-sided obstruction defects, and septation defects.

## 3. microRNAs in Cyanotic Heart Diseases

Cyanotic heart diseases encompass defects such as TGA, TOF, tricuspid atresia, pulmonary atresia, Ebstein’s anomaly, DORV, PTA and total anomalous pulmonary venous connection. To date, involvement of microRNAs on these cardiac pathologies has only been described for TGA, TOF and DORV.

TGA, also referred to as complete transposition, is a congenital cardiac malformation characterized by atrioventricular concordance and ventriculoarterial discordance. The incidence is estimated at 1 in every 3500–5000 births having an increased ratio on males. While our current understanding of the functional role of microRNAs in TGA is still in its infancy, seminal studies have provided evidence that distinct microRNAs might serve a biomarkers of adult TGA such as those reported by Lai et al. [51], yet Tutarel et al. [52] failed to find such differences in their study. Therefore, additional studies are required to clarify such controversy.

TOF is the most common cause of cyanotic cardiac disease in patients beyond the neonatal age, accounting for >10% of all congenital cardiac lesions [53], and with a prevalence of approximately 5 per 10,000 live births. TOF is a clinical entity combining anatomic malformations such as interventricular communication, biventricular connection of the aortic root, obstruction of the right ventricular outflow tract and right ventricular hypertrophy [54]. The degrees of right ventricular outflow tract (RVOT) obstruction widely varies and the presence of large VSD normally requires surgical intervention within the first years of life [53,54].

Several studies have provided evidence of genetic associations between distinct microRNA single nucleotide variants and the occurrence of TOF, in particular on miR-17 [55] and miR-196 [56]. However, evidence of their functional implications is still lacking. On the other hand, a large dataset has provided evidence of the involvement of multiple deregulated microRNAs in distinct conditions of TOF, including pediatric [14,57,58,59,60,61] and corrected adult patients [62,63].

**Figure 1:**
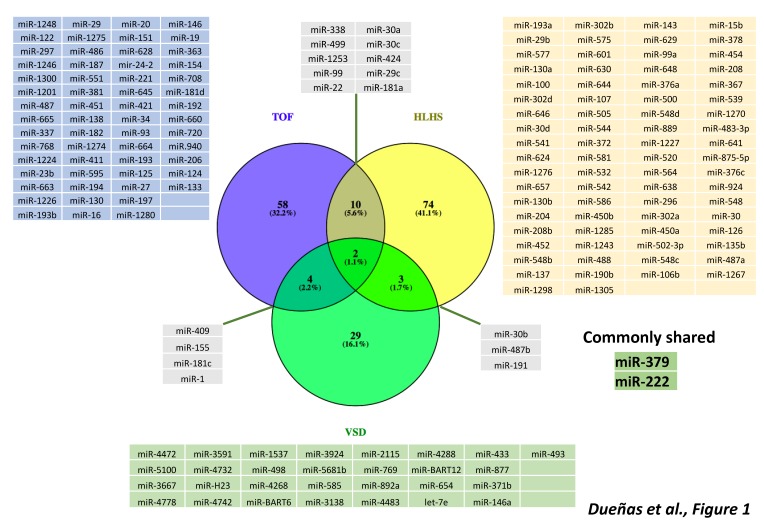
Schematic representation of distinct microRNAs involved in TOF (Tetralogy of Fallot), HLHS (Hypoplastic Left Heart Syndrome) and VSD (Ventricular Septal Defect), as reported by distinct differential gene expression analyses. Observe that the majority of the differentially expressed microRNAs are associated to a single CHD entity and only 1.1% are shared among all three CHDs, i.e., miR-379 and miR-222.

In seminal studies, O’Brien et al. [57] identified 61 microRNAs that display significant changes in expression levels in children with TOF compared with controls. Interestingly, the levels of microRNAs expression in children with TOF remain similar to those in the normal fetal myocardium. The authors identified 33 microRNAs that were significantly downregulated in TOF myocardial tissue compared to the normally developing myocardium. Zhang et al. [58] also described 18 differentially expressed microRNAs in TOF patients, and demonstrated the role of miR-424 and miR-222 regulating cardiomyocyte proliferation and thus a plausible role on TOF development. Analyses of microRNA and mRNA complementary expression identified the functional relationship between miR-421 and SOX4 expression in TOF patients, as described by Bittel et al. [59]. SOX-4 is a key regulator of the Notch pathway, suggesting miR-421-SOX4 signaling might be a potential contributor to TOF development [59]. Moreover, of those 44 genes identified in TOF cardiac network, 51% are critical for cardiac development and importantly splice variants mediated by snoRNAs were also identified. Overall, these data suggest that impaired expression of ncRNAs, both microRNAs and snoRNAs, may contribute to the development of CHD, particularly to TOF, by influencing expression, differential transcript splicing and translation of genes and/or proteins that are important for normal heart development [39] (Figure 1).

Additional evidence of the functional role of microRNAs in TOF pathogenesis were described by Liang et al. [64], who identified that miR-940 is highly expressed in the normal human RV outflow tract but dramatically down-regulated in TOF patients. Functional analysis revealed that miR-940 downregulation blocked cardiac resident cell migration by targeting *JARID2*, a gene previously involved in cardiac outflow tract development. Furthermore, miR-1 and miR-206 were significantly down-regulated in TOF patients and both microRNAs impaired Cx43 expression, suggesting that both microRNAs may contribute to the development of TOF, since Cx43 loss of function leads to conotruncal anomalies [59]. Additional evidence on the role of microRNAs in pediatric cases of TOF were provided by Wang et al. [60], who described a microRNA–mRNA genetic network, in which miR-124 and miR-138 play pivotal roles. Furthermore, Wang et al. [61] discovered sexual differences in microRNA expression of TOF patients, supporting a key role to miR-1 and miR-133. Besides these findings in pediatric cases of TOF, microRNAs seem to also play a key role on TOF-related cardiac pathophysiology in adulthood (Figure 1). To date, two reports provide evidence on microRNA differential expression in adult TOF patients [62,63], yet whether they can serve a bona fide biomarkers of disease evolution remains controversial. Despite increasing evidence on differentially expressed microRNAs in TOF patients, the functional contribution of individual microRNAs remains to be elucidated.

DORV is present when both the aorta and pulmonary trunk originate, predominantly or entirely, from the right ventricle [65]. In this situation, the left ventricle has no direct outlet to a great artery and thus ejects through the interventricular communication, since VSD is almost invariably present. Cyanosis is frequent but not universal. DORV results from impaired formation of the ventricular outlet during early embryonic life [65]. While there are no reports of the implication of microRNAs in DORV in humans, experimental evidence in mice unraveled the fundamental role of microRNAs in DORV development. Conditional deletion of *DICER* in either the developing embryonic heart [66] or specifically in the cardiac neural crest cells [67] leads to DORV.

## 4. microRNAs in Left-Sided Cardiac Obstructions

Left-sided obstructive lesions, the second main type of CHD, include HLHS, mitral stenosis, aortic stenosis, aortic coarctation and IAA. To date, involvement of microRNAs in these cardiac pathologies have only been described for HLHS.

HLHS is defined as underdevelopment of the structures of the left side of the heart, which includes the mitral valve, left ventricle, aortic valve, and aortic arch. The absence of atrial communication in HLHS produces severe obstruction of pulmonary blood flow frequently causing vascular injury [68]. Despite the relatively low incidence, it is responsible for 23% of cardiac deaths occurring in the first week of life [69].

In a recent study by Sucharov et al. [70], the microRNA profile of the RV in HLHS patients was compared with the RV of non-failing hearts. A total of 93 microRNAs differentially regulated in HLHS hearts. In a mouse model, some of these microRNAs have been implicated in the fetal program during heart failure (miR-208) and in RV hypertrophy and failure (miR-30 and miR-378). Additionally, three microRNA species (miR-99a, miR-100 and miR-145a) were down regulated prior to or directly after stage 1 operation of Norwood procedure while values returned to control levels after stage 3 operation, demonstrating strong influence of altered blood flow conditions on microRNA expression. miR-137 and miR-145, which are down-regulated in the RV of HLHS patients, directly regulate the expression of *FOG-2*, a transcription factor that modulates the expression of *GATA-4*, *GATA-5* and *GATA-6*, essential players in cardiac development (Figure 1). In contrast, miR-204 is up-regulated in the hypertrophic RV of HLHS patients, similar to patients who suffer from hypertrophic cardiomyopathy. Thus, to date, our current understanding of the functional role of microRNAs in HLHS is still incipient and additional studies are required to dissect their function in HLHS.

## 5. microRNAs in Septal Defects

Septation defects, the third main type of CHD, can affect separation of the atria (ASDs), the ventricles (VSDs) or both (AVSDs) [71,72,73]. Most children with isolated ASDs are free of symptoms, but rates of exercise intolerance, atrial tachyarrhythmias, right ventricular dysfunction, and pulmonary hypertension increase with age and life expectancy is reduced in adults with untreated defects [74,75]. The risk of development of pulmonary vascular disease, a potentially lethal complication, is higher in female patients and in older adults with untreated defects [74,75]. More than 50% of CHDs are caused by sporadic ASD or VSD [76,77]. To date, involvement of microRNAs in these cardiac pathologies have only been described for VSD, while several studies reported distinct microRNAs as biomarkers of ASD [78], VSD [79] and AVSD [80]. In addition, polymorphisms in different microRNAs, such as miR-196, miR-27, miR-499 have been associated to distinct CHDs, particularly septal defects [81] and polymorphisms in ACTA1 3´UTR, affecting miR-139 regulation, has been recently reported to association to the occurrence of ASD [82].

VSD is one of the commonest congenital malformations of the heart, accounting for up to 40% of all cardiac anomalies. This is not only a common isolated cardiac malformation but also an intrinsic component of several complex malformations, including TOF or univentricular atrioventricular connection. Failure of complete formation of the primitive interventricular septum or failure of fusion of the atrioventricular cushions with each other or with the primary septum are common developmental defects leading to VSD [83,84].

Li et al. [15] detected 36 microRNAs dysregulated expression in circulating plasma of VSD patients. Seven of these microRNAs, namely, let-7e, miR-155, miR-222, miR-379, miR-409, miR-433 and miR-487, were down-regulated while miR-498 was up-regulated (Figure 1). Gene ontology analysis revealed *NOTCH1, HAND1, ZFPM2* and *GATA3* as putative targets, genes known to be involved in right ventricle morphogenesis. miR-1 and miR-181c have been implicated in VSD pathogenesis. miR-1 is a regulator of *BMP1* while miR-181c can regulate *SOX9*. In human cardiac tissue with VSD, elevated levels of *GJA1* and *SOX9* coincided with reduced expression of miR-1, and elevated miR-181c expression was associated with down regulation of *BMPR2* [85]. Therefore, dysregulation of these genes by these microRNAs might contribute to development of VSD.

Gene-targeting approach of miR-1-2 results in heart defects that include VSDs. Surviving mice have conduction-system defects and increased cardiomyocyte proliferation [41,86]. In addition, Smith et al. [87] showed that miR-1 knockouts display a reduced pool of proliferating ventricular cardiomyocytes in the developing heart. Furthermore, haploinsufficiency of miR-1 and miR-133a is associated with an increased risk of VSD via a process of *HAND2* and *SRF* regulation, respectively.

miR-133a-1 and miR-133a-2 genes are both expressed in the linear heart tube and subsequently in the cardiac chambers during mouse embryogenesis [88,89]. Singular deletion of either microRNA in mice does not result in pathology, however combined deletion of both genes produces late embryonic and neonatal deaths due to VDS and dilatation of the cardiac chambers.

Targeted deletion of the miR17-92 family of microRNAs results in the neonatal lethality from lung hypoplasia and VSDs [48]. This cluster and its paralog clusters, miR-106a-363 and 106b-25, belong to a family of highly conserved microRNA clusters that consists of six members: miR-17, miR-18a, miR-19a, miR-20a and miR-19b-1 and miR-92a-1. Knockout mice that lacked the paralog clusters miR-106a-363 and miR-106b-25, respectively, did not display an obvious phenotype, whereas mice with a loss of both clusters developed exacerbated cardiac failure, including ventricular wall thinning and VSD [90].

Another microRNA involved in the formation of VSDs is miR-195, a member of the miR-15 family. Early cardiac muscle-specific overexpression of miR-195 under control of the β-myosin heavy chain promoter during embryogenesis was associated with ventricular hypoplasia and VSDs [39,91]. Additional evidence on the role of microRNAs in CHDs is provided by conditional deletion of *DGCR8*, an RNP essential for microRNA biogenesis, in cardiac neural crest cells, resulting in PTA and VSD [92]. Overall, these studies unraveled several microRNA candidates that might play a pivotal role in VSD.

## 6. Long Non-Coding RNAs (lncRNAs) in Congenital Heart Diseases

Long non-coding RNAs (lncRNAs) are a subclass of non-coding RNA transcripts longer than 200 nucleotides. LncRNAs are stable in plasma and urine and display tissue and disease specificity. To date, more than 3000 lncRNAs have been identified and one-third of them have already been found to be involved in various biological processes, including cell growth, differentiation, cell proliferation and apoptosis [31,32,36,37]. Recent studies suggest that circulating plasma lncRNAs have great potential as new diagnostic and prognostic biomarkers and play important roles in effective evaluation of treatments in disease such as cardiovascular disease [93] and cancer [94,95,96]. lncRNAs display a wide variety of biological functions including both transcriptional and post-transcriptional regulation in multiple cellular processes as detailed below [97,98].

At transcriptional level, at least four different types of actions have been demonstrated: (a) lncRNAs act as guide, binding to transcription factors or protein subunits of chromatin remodeling complexes and driving them towards their genomic targets. Thus, lncRNAs can promote or suppress gene activity depending on whether the guided complexes are activating, such as MLL complexes, or repressing, such as PRC2 complexes [99]. (b) Scaffold lncRNAs can act at different complexes facilitating their assembly and being a functional component of it [100,101]. (c) Enhancer lncRNAs can act as enhancers of transcription, promoting and maintaining the genomic 3D conformation required for transcriptional machinery to get access to promoter regions [102,103]. (d) The last class of transcriptional regulatory lncRNAs exert their function as decoy molecules competing with transcription factors or chromatin remodeling complexes for their genetic targets [104,105].

At post-transcriptional level lncRNAs can provide at least three different types of action, interacting with mRNAs, translational machinery or other non-coding RNAs such as microRNAs [106]. Nuclear lncRNAs participate on pre-mRNA maturation by interacting with pivotal alternative splicing factors [107]. For example, sno-lncRNAs located in the nucleolus and Cajal bodies can associate with *FOX* proteins, such as *FOX2* and regulate mRNA alternative splicing [108]. lncRNAs can affect mRNA stability by base-pairing with them and altering their half-lives. Incomplete base-pairing normally promotes mRNA decoy whereas a full base pairing between both usually promotes mRNA stability and thus protein translation [109,110,111]. Interestingly many lncRNAs are associated with ribosomes and can therefore affect mRNA translation, interacting with the 3′UTR region and modulating the assembly of translation initiation complex [106,112]. Finally, several lncRNAs can act upon microRNAs interacting with them and serving as microRNA sponges, thus modulating post-transcriptional gene expression [113,114].

Tissue specificity of lncRNAs was previously based on differential expression patterns in specific biological systems. Due to the limited information available about the function of each lncRNA and the lack of strong conservation, this fact constitutes a strong set back, but in contrast lncRNA expression tends to be spatially and temporally restricted [115,116]. One of the main challenges for ncRNA research is the identification of an association with a particular molecular or cellular function. Recent evidence suggests that lncRNAs acts locally, regulating the expression levels of neighboring RNA transcripts [117], however, physical proximity of lncRNAs and genes with developmental functions does not necessarily imply a functional link between the protein coding gene and the lncRNA. The main reason for this is that lncRNAs may employ varied mechanisms of action that not always imply *cis–trans* way.

**Figure 2:**
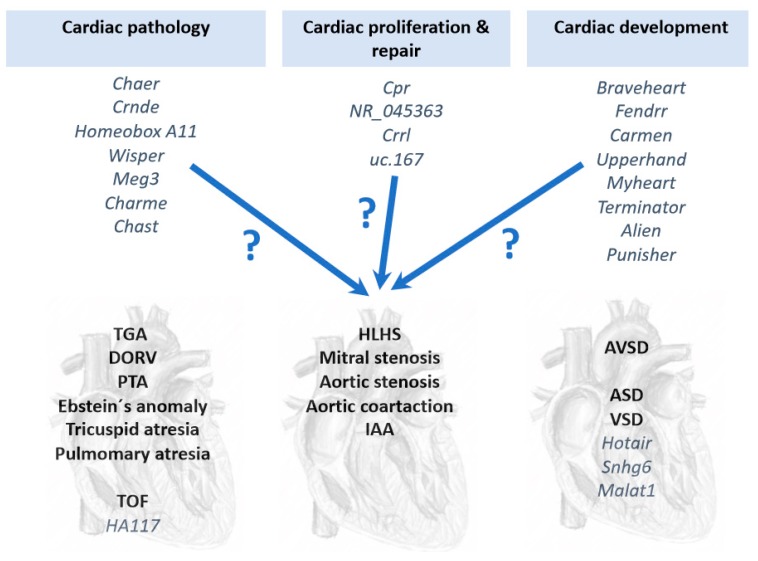
Schematic representation of distinct lncRNAs involved cardiac pathology, cardiomyocyte cell proliferation and repair and cardiac development, which might have functional contribution to CHD. At present, only a minor subset of lncRNAs have been associated to CHD, particularly HA117 in TOF and Hotair, Sngh6 and Malat1 in VSD.

Several lcnRNAs have been reported to play fundamental role in distinct cardiac pathological conditions, such as *Myheart* and *Chaer* in cardiac hypertrophy [105,118]; *Crnde, Homeobox A11, Wisper* and *MEG3* in cardiac fibrosis [119,120,121,122]; and *Charme* and *Chast* in cardiac remodeling [123,124] (Figure 2). In addition, several lncRNAs have also been reported to enhance cardiomyocyte proliferation and repair, such as *Cpr* [125], *NR_045363* [126], *Crrl* [127] and *uc.167* [128] (Figure 2), yet to date none of these lncRNAs have been associated to CHDs. Furthermore, several lncRNAs have been implicated in key developmental processes related to cardiac development, such as *Braveheart* [129,130], *Fendrr* [99,131], *Carmen* [132], *Upperhand* [133], *Terminator, Alien* and *Punisher* [134] (Figure 2). Similar to those reported in cardiac pathophysiology, no functional linkage to CHD has been reported to those lncRNAs involved in cardiac development. Current information of the functional role of lncRNAs in CHDs is thus scarce and only a short list of reports have provided evidence of the involvement of lncRNAs in CHDs, particularly on VSD and TOF. Gu et al. [93] identified differentially expressed lncRNAs which have potential diagnostic value in predicting fetal CHD, providing evidence that circulating plasma lncRNA may serve as novel biomarkers for CHD diagnosis. Jiang et al. [135] reported increased HOTAIR expression in right atrial biopsies of patients with CHD, primarily ASD and VSD, postulating HOTAIR as a biomarker for CHD.

Jiang et al. [135] reported increased SNHG6 expression in fetal cardiac tissues of VSD patients. Experimental analyses demonstrate that SNHG6 gain-of-function blocked cardiomyocyte proliferation while enhanced apoptosis. In addition, miR-101 was downregulated and Wnt/β-catenin pathway was activated, suggesting a plausible mechanistic link between SNHG6 up-regulation, impaired miR-100 expression, Wnt/β-catenin activation and the formation of VSD.

Song et al. [136] conducted a study regarding the dysregulated lncRNAs in VSD-affected cardiac tissue. Among them, 905 were intergenic, 201 intronic antisense, 175 natural antisense, 61 bidirectional, 38 exon sense and 28 intron sense-overlapping sequences, yet their functional implication in VSD development remain unsolved. More recently, Li et al. [35] identified a MALAT1 polymorphism associated to CHDs, particularly to VSD and ASD. Overall, these studies enlighten the importance of lncRNAs in cardiac septal defects.

TOF is a severe CHD that leads to numerous complications and poor outcomes in several patients. *HA117* is a lncRNA related to cell differentiation and later found to display anti-differentiation functions in leukemia cells, solid tumors and Hirschsprung’s disease. *DPF3* and *RGS6* are neighboring genes to *HA117* and play a fundamental role in the heart. Wang et al. [137] postulated a relationship between TOF and *HA117*, since high *HA117* expression is associated to adverse outcomes in TOF patients. To date, the mechanism of action of *HA117* in TOF remains unclear. As in the case of septal defects, the study by Wang et al. [137] is seminal, supporting a role of lncRNAs in TOF development, yet additional studies are required to fully elucidate the functional relevance of lncRNAs in this context.

## 7. Conclusions and Perspectives

Seminal studies have provided evidence that transcriptional regulation is pivotal for correct cardiovascular development and that, if impaired, congenital heart defects are prone to occur. In addition to transcriptional regulation of the developing cardiovascular system, increasing evidence demonstrates a substantial contribution of post-transcriptional mechanisms, such as those driven by non-coding RNAs. Experimental genetic manipulation of several microRNAs led to CHDs such as DORV and VSD highlighting the importance of these non-coding RNAs in cardiogenesis. Seminal studies in TGA patients were able to identify microRNAs as biomarkers supporting their use as predictive tools. Furthermore, a bulk of studies also demonstrate differential expression of microRNA associated to distinct CHD, such as TOF, HLHS and VSD, opening up the possibility that many more microRNAs will be instrumental in the occurrence of CHDs. Importantly, a comparison between those differentially expressed microRNAs identified in TOF, HLHS and VSD results in minimal overlap (Figure 1), suggesting that distinct microRNA molecular hallmark applies to each CHD. In other words, a distinct subset of microRNAs is involved in each CHD, supporting highly specific role from distinct microRNAs during cardiac development (Figure 1). While increasing evidence is emerging on the functional role of lncRNAs in cardiac development and disease, identification of the role of lncRNAs in CHDs is still incipient (Figure 2), in part because their complexity, both structurally and functionally. In next coming years, we will witness an explosion of studies that will support the increasing functional role of non-coding RNAs in the onset of CHDs.
